# Circulating levels of matrix proteases and their inhibitors in pregnant women with and without a history of recurrent pregnancy loss

**DOI:** 10.1186/1477-7827-8-62

**Published:** 2010-06-16

**Authors:** Dilly OC Anumba, Saad El Gelany, Sarah L Elliott, Tin C Li

**Affiliations:** 1 Section of Endocrinology and Reproduction, Academic Unit of Reproductive and Developmental Medicine, University of Sheffield, 4th Floor, Jessop Wing, Tree Root Walk, Sheffield S10 2SF, UK

## Abstract

**Background:**

We have recently shown that serum relaxin-2 levels are attenuated in women with a history of recurrent pregnancy loss (RPL). We sought to determine whether a history of RPL is also associated with changes in serum matrix metalloproteases (MMPs) and tissue inhibitors of matrix metalloproteases (TIMP) -1 and -2.

**Methods:**

We obtained serum from 20 pregnant women with a history of RPL and 20 age-matched pregnant women with no history of RPL (NRPL) at 6-8, 10-12, 20, and 34 weeks gestation, and from cord blood. We quantified total serum concentrations of MMP-1, MMP-3, MMP-9 and TIMP-1 and TIMP-2 by ELISA. We determined whether these serum marker levels were associated with a history of RPL and delivery before 37 weeks gestation.

**Results:**

There was no difference in the rates of miscarriage, preterm birth or prelabour rupture of fetal membranes between RPL and NRPL. However babies born to RPL were lighter than those born to NRPL. Serum MMP-1, 9, and TIMP-1 did not differ between RPL and NRPL but MMP-3 was higher in RPL vs. NRPL at 6-8 weeks (P < 0.05). Serum TIMP-2 levels were higher in RPL women at all gestations (P < 0.01). The ratio of RLX-2 (reported previously) to TIMP-2 at 10-12 weeks gestation was more strongly associated with a history of RPL than either peptide separately - area under the ROC curves for RLX-2 0.79 (95% CI 0.57 to 0.92), TIMP-2 0.83 (95% CI 0.63 to 0.95), and for RLX-2:TIMP-2 ratio 0.92 (95% CI 0.74 to 0.99).

**Conclusions:**

Women with a history of RPL demonstrate increased serum TIMP-2 and reduced RLX-2 during a subsequent viable pregnancy. Determination of both markers in early pregnancy enhances the discrimination of women with a history of RPL. These observations suggest roles for these two peptides in early implantation and placental development. Whether these may prove to be reliable early predictive markers for subsequent pregnancy loss in the index pregnancy is unknown and will require further studies.

## Background

Recurrent pregnancy loss (RPL) is a distressing clinical problem and affects 1% of all women. Although some of the associated conditions are known [1-4], the aetiology remains poorly understood and the course of any future pregnancy remains uncertain. Several serum factors are altered in some women with RPL but none of them reliably predicts repeat miscarriage [5-8]. Insulin resistance characterises RPL associated with the polycystic ovary (PCOS) syndrome but is not a reliable marker for repeat pregnancy loss [3].

We have recently demonstrated that serum Relaxin-2 (RLX-2) levels are attenuated in women with a history of RPL [9] and correlate with uterine artery Doppler resistance parameters in first trimester, consistent with a crucial role for this peptide in implantation and placental development [10,11]. Higher rates of adverse pregnancy outcomes associated with uteroplacental insufficiency have been reported in women with a history of RPL [12].

Matrix metalloproteinases (MMPs) are a family of proteolytic enzymes that play a central role in the breakdown and reorganization of extracellular matrix. The group consists of > 20 members and includes collagenases (MMP-1, -8, and -13), stromelysins (MMP-3, -7, and -10), and gelatinases (MMP-2 and -9). Tissue inhibitors of matrix metalloproteinases (TIMPs) metabolise MMPs and relaxin has been noted to regulate MMP and TIMP activity in several tissues [13-15]. Together with relaxin [16], MMPs and TIMPs appear to play some roles in embryo implantation, trophoblast invasion, early placentation, and cervical dilatation and feto-maternal membrane lysis in later gestation [16,17]. Importantly, the secretion of MMPs and TIMPs by trophoblast cells is regulated by the composition of the surrounding extracellular matrix [18]. Consistent with these roles, we hypothesized that women with a previous history of RPL may demonstrate altered expression levels of some MMPs and TIMPs, similar to our observations for RLX. We compared the serum concentrations of interstitial collagenase (MMP-1), stromelysin-1 (MMP-3), gelatinase B (MMP-9), TIMP-1 and TIMP-2 in aliquots of serum stored from women with a history of RPL and age-matched pregnant controls without a previous history of miscarriage at several gestational time points.

## Methods

### Subjects

Frozen aliquots of serum obtained from 20 pregnant women with a history of RPL (defined as the loss of three or more consecutive pregnancies before 24 weeks) and 20 age-matched pregnant controls with no previous history of RPL or an identified association with RPL, stored at -80°C, were assayed for MMP-1, -3, -9, and TIMP-1 and -2. Serum samples had been obtained and aliquoted at 6-8, 10-12, 20, and 34 weeks gestation during a previous study [9]. We excluded women who smoked cigarettes from the study. Sample sizes were determined as previously described [9]. We estimated that the 20 subjects in each study arm would detect a 10% difference in MMP and TIMP levels between groups with 80% power at the 95% confidence level. Written informed consent was obtained from each study participant. Ethical permission was obtained from the South Sheffield Research Ethics committee.

### Study design

The protocol and time points for serum sample collection and preparation were as previously reported [9]. At ≥ 10 wks, fetal biometry and uterine artery Doppler indices were simultaneously measured by transabdominal ultrasound [16].

### Laboratory analysis

Serum MMP-1, -3, and -9 and serum TIMP-1 and -2 total concentrations (free and protein bound fractions) were measured using enzyme immunoassay kits (Catalog Numbers QIA55, QIA56, QIA73, QIA54, QIA40 respectively, Callbiochem, San Diego, USA) according to the manufacturer's instructions. The assays were based on the quantitative sandwich enzyme technique. Test samples and standards were pipetted onto the surface of the plastic wells containing monoclonal antibodies specific for the respective MMP or TIMP provided in the kit. The specific human protein present thus got bound to the capture antibody. Unbound material was then washed away and a monoclonal, horseradish peroxidase (HRP)-conjugated specific anti-MMP or anti-TIMP antibody was added to the wells, followed by a chromogenic substrate. The horseradish peroxidase catalysed the conversion of a chromogenic substrate from a colorless solution to a blue solution, the intensity of which was proportional to the amount of human MMP or TIMP protein in the test sample. The coloured reaction product was then quantified using a spectrophotometer by the construction of a standard curve using known concentrations of the respective human MMP or TIMP protein (provided lyophilized) as internal controls. All the kits for all the analytes were highly precise, with inter- and intra-assay coefficients of variation < 10%.

### Doppler studies

The rationale for, and assessment of, maternal uterine artery (UA) Doppler velocimetry have been described previously [9,19]. The resistance index (RI) has been shown to be the most repeatable and least variable of the UA Doppler indices. We therefore employed the average RI of the left and right UA Doppler waveform for tests of association. All clinical details, including the pregnancy outcome for both groups, were recorded.

### Outcome measures

We determined whether MMP and or TIMP levels a) better separated women with a history of RPL from those without; b) demonstrated any correlation with serum RLX-2 or UA RI indices and c) were associated with spontaneous miscarriage, delivery < 37 weeks, small for gestational age (birth weight < 10th customized centile for gestation [20]), placental abruption or stillbirth.

### Data analysis

Serum MMP and TIMP levels were compared by one-way ANOVA, the student t-test (for normally distributed data) or the Mann-Whitney U test (for data not normally distributed) with appropriate post hoc correction (the Bonferroni test) for repeated measures and multiple comparisons. Binary logistic regression analyses and the area under the Receiver Operator Characteristic (AuROC) curves were used to test association between MMP and TIMP levels and categorical clinical outcome variables. Where data regarding serum markers were missing a listwise deletion approach was adopted for analysis.

## Results

The clinical details of study participants and the outcome of their pregnancies have been published previously [9], and only details pertinent to this report are summarized in Table 1. Of the 20 participants with a history of RPL, 12(60%) had idiopathic RPL, 5(25%) had the APS, and 3(15%) had the PCOS associated with suspected luteal phase deficiency. All those with the APS received low molecular weight heparin (LMWH) and low-dose (75 mg) aspirin throughout pregnancy. There were no stillbirths in either group and only one miscarriage in the RPL group. There was no significant difference in maternal body mass index, pregnancy duration, rates of preterm birth and prelabour rupture of fetal membranes between study groups and between aetiological association subtypes in women with RPL.

**Table 1:** Subject characteristics, pregnancy outcome, and serum levels of MMPs and TIMPs, pregnant women with a history of RPL vs. pregnant women with no history of RPL

	History of recurrent pregnancy loss, RPL	No history of recurrent pregnancy loss, NRPL	P value
n	20	20	
Median (range) age (yrs)	32.5 (20 - 43)	31.0 (24-41)	0.8
Mean (SE) birth weight (g)	3040 (206)	3364 (216)	0.15
Infant birth weight < 2500 g, n (%)	7 (35)	3 (15)	0.15
Small for gestational age (birth weight < 10^th ^centile)	5 (25%)	0	0.048
**Mean (SE) serum MMP-1 levels in ng/ml**			
6-8 wks	346 (36)	438 (104)	0.35
10-12 wks	355 (52)	366 (55)	0.69
20 wks	337 (29)	380 (71)	0.55
34 wks	524 (52)	529 (104)	0.97
Cord serum	375 (41)	476 (115)	0.36
**Mean (SE) serum MMP-3 levels in ng/ml**			
6-8 wks	5.6 (0.7)	3.2 (0.4)	0.01
10-12 wks	7.4 (2.4)	5.2 (0.7)	0.39
20 wks	5.3 (0.7)	5 (0.7)	0.74
34 wks	4.7 (0.6)	5 (0.6)	0.64
Cord serum	1.8 (0.4)	1.8 (0.4)	0.90
**Mean (SE) serum MMP-9 levels in ng/ml**			
6-8 wks	70.0 (6.9)	86.0 (15.3)	0.31
10-12 wks	91.5 (10.3)	83.3 (13.0	0.62
20 wks	105.8 (12.4)	80.6 (12.8)	0.15
34 wks	89.4 (11.3)	111.8 (14.6)	0.23
Cord serum	47.6 (8.7)	51.7 (7.4)	0.72
**Mean (SE) serum TIMP-1 levels in ng/ml)**			
6-8 wks	20.0 (2.0)	17. 1 (2.4)	0.37
10-12 wks	23.7 (1.8)	20.6 (3.5)	0.42
20 wks	23.9 (2.8)	23.3 (2.7)	0.88
34 wks	18.4 (2.1)	18.9 (2.4)	0.87
Cord serum	17.9 (2.7)	21.7 (3.0)	0.35
**Mean (SE) serum TIMP-2 levels in ng/ml**			
6-8 wks	36.0 (8.4)	13.8 (1.4)	0.01
10-12 wks	31.0(5.2)	11.7 (1.1)	0.002
20 wks	23.6 (5.2)	9.9 (0.9)	0.008
34 wks	23.7 (3.2)	16.9 (1.3)	0.05
Cord serum	16.7 (6.1	11.1 (1.1)	0.44
Relaxin:TIMP-2 ratio*			
6-8 wks	0.05 (0.01)	0.18 (0.03)	0.006
10-12 wks	0.05 (0.01)	0.17 (0.03)	0.0001
20 wks	0.03 (0.01)	0.08 (0.02)	0.03
34 wks	0.04 (0.01)	0.13 (0.02)	0.02
Cord serum	0.01 (0.01)	0.00 (0.00)	0.13

* Relaxin levels were derived from an earlier assay [9].

**Figure 1:**
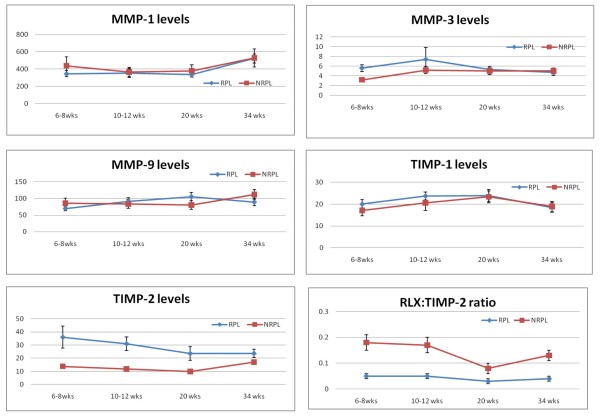
Serum concentrations of matrix proteins and their inhibitors in study groups. Comparison of serum concentrations (ng/ml) of MMP-1, MMP-3, MMP-9, TIMP-1, TIMP-2, and ratio of RLX to TIMP-2, RPL vs NRPL, * P < 0.05, ** P < 0.01.

**Figure 2:**
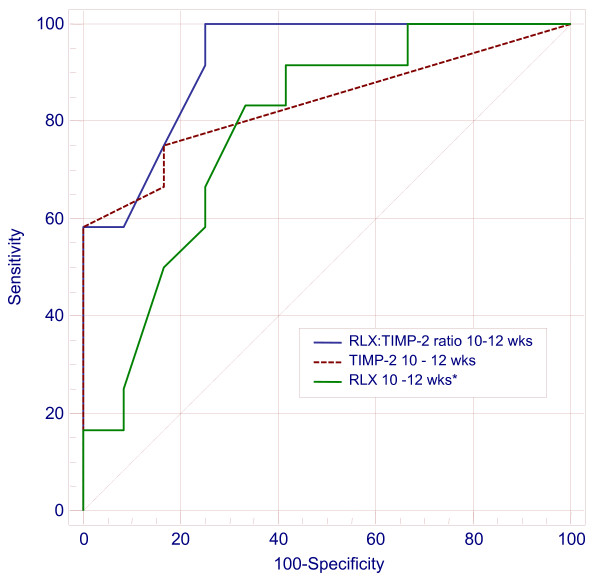
Association of relaxin and TIMP-2 with a history of RPL. ROC curves comparing association of RLX-2, TIMP-2 and RLX-2:TIMP-2 ratios at 10-12 weeks gestation in women with a history of RPL (*vs. RLX-2:TIMP-2, P < 0.05).

Serum MMP-1, -3, -9 and TIMP-1 and -2 levels in our study groups did not differ with maternal gravidity or parity (Table 1). MMP-1, MMP-9, and TIMP-1 did not differ between RPL and NRPL groups at all study gestations, and between aetiological association subtypes in the women with RPL. Serum levels of MMP-3 were higher in RPL compared to NRPL at 6-8 weeks gestation (P < 0.05) but did not differ at other gestational time points. In contrast, TIMP-2 levels were significantly higher in women with a history of RPL compared to NRPL at all gestational time points (Table 1, Figure 1), whilst umbilical cord serum levels of all study proteins did not differ. Furthermore, expressing serum RLX-2 (reported in an earlier assay [9] and TIMP-2 levels as a ratio improved the strong individual associations with a history of RPL, increasing the AuROC curves at all study gestations (Table 2). Comparison of AuROC curves showed that the stronger association of low RLX-2:TIMP-2 ratios with a history of RPL attained statistical significance at 10-12 weeks gestation, compared to RLX-2 levels alone (P < 0.05, Figure 2).

**Table 2:** Area under the ROC curves (AuROC) and associations between serum Relaxin, TIMP-2 and relaxin:TIMP-2 ratios and a history of RPL

	AuROC (95% CI)	P value	Optimal predictive cut-off value	Sensitivity (95% CI)	Specificity (95% CI)	+LR (95% CI)	-LR (95% CI)
**6-8 wks gestation**							
Relaxin (ng/ml)	0.65 (0.50 - 0.80)	< 0.05	< = 1.3	80.00 (56.3 - 94.3)	46.67 (21.3 - 73.4)	1.50 (0.8 - 2.7)	0.43 (0.2 - 1.2)
TIMP-2 (ng/ml)	0.84 (0.60 - 0.97)	< 0.001	> 14	77.78 (40.0 - 97.2)	70.00 (34.8 - 93.3)	2.59 (1.5 - 4.4)	0.32 (0.07 - 1.5)
Relaxin:TIMP-2 ratio	0.89 (0.67-0.99)	< 0.0001	<= 0.06	77.78 (40.0 - 97.2)	90.00 (55.5 - 99.7)	7.78 (5.2 - 11.7)	0.25 (0.03 - 2.3)
**10-12 wks gestation**							
Relaxin (ng/ml)	0.73 (0.54 - 0.90)	< 0.05	<= 1.7	95.00 (75.1 - 99.9)	42.11 (20.3 - 66.5)	1.64 (1.0 - 2.8)	0.12 (0.02 - 0.8)
TIMP-2 (ng/ml)	0.83 (0.63 - 0.95)	< 0.001	> 10	75.00 (42.8 - 94.5)	83.33 (51.6 - 97.9)	4.50 (3.0 - 6.8)	0.30 (0.06 - 1.5)
Relaxin:TIMP-2 ratio	0.92 (0.74 - 0.99)	< 0.0001	<= 0.09	100.00 (73.5 - 100.0)	75.00 (42.8 - 94.5)	4.00 (2.9 - 5.5)	0.00 (0.01 - 0.9)
**20 weeks gestation**							
Relaxin (ng/ml)	0.77 (0.58 - 0.90)	< 0.01	<= 0.71	78.95 (54.4 - 93.9)	61.11 (35.7 - 82.7)	2.03 (1.3 - 3.1)	0.34 (0.1 - 1.0)
TIMP-2 (ng/ml)	0.73 (0.52 - 0.88)	< 0.05	> 20	50.00 (21.1 - 78.9)	100.00 (78.2 - 100.0)		0.50
Relaxin:TIMP-2 ratio	0.81(0.61-0.93)	< 0.001	<= 0.05	66.67 (34.9 - 90.1)	80.00 (51.9 - 95.7)	3.33 (2.1 - 5.4)	0.42 (0.1 - 1.5)
**34 weeks gestation**							
Relaxin (ng/ml)	0.68 (0.48 - 0.84)	0.13	<= 0.9	94.44 (72.7 - 99.9)	47.06 (23.0 - 72.2)	1.78 (1.1 - 3.0)	0.12 (0.02 - 0.8)
TIMP-2 (ng/ml)	0.67 (0.46 - 0.85)	0.11	>14	66.67 (34.9 - 90.1)	30.77 (9.1 - 61.4)	0.96 (0.4 - 2.4)	1.08 (0.5 - 2.6)
Relaxin:TIMP-2 ratio	0.77 (0.56- 0.92)	<0.01	<= 0.04	83.33 (51.6 - 97.9)	76.92 (46.2 - 95.0)	3.61 (2.4 - 5.3)	0.22 (0.04 - 1.1)
**Umbilical cord levels**							
Relaxin (ng/ml)	0.52(0.32 - 0.71)	NS					
TIMP-2 (ng/ml)	0.50 (0.28 - 0.72)	NS					
Relaxin:TIMP-2 ratio	0.56 (0.32-0.78)	NS					

NS: not significant.

At 10-12 wks gestation a RLX-2:TIMP-2 ratio ≤ 0.1 was best associated with a history of RPL [sensitivity 100%, specificity 75%, LR+ 4.0 95% CI 2.9 - 5.5, LR- 0.00 95% C1 0.01 - 0.9, P < 0.0001), Table 2]. At 20 wks gestation RLX-2:TIMP-2 ratio ≤ 0.05 was best associated with a history of RPL [sensitivity 67%, specificity 80%, LR+ 3.3 95% CI 2.1 - 5.4, LR- 0.42 95% C1 0.1 - 1.5, P < 0.001), Table 2] Among women with RPL serum MMP and TIMP levels did not differ by cause of miscarriage or by treatment with aspirin or LMWH (data not shown).

Stepwise logistic regression analysis (factors included in the model: maternal age and body mass index, RLX-2:TIMP2 ratios at study gestations) demonstrated that the RLX-2:TIMP-2 ratios in RPL were lower than in NRPL group at 10-12 wks gestation (regression coefficient -0.43, SE 0.2, OR 0.65, 95% CI 0.44, 0.97, P < 0.0001), at 20 wks gestation (regression coefficient -2.0, SE 0.1, OR 0.80, 95% CI 0.65, 0.98, P < 0.01), and at 34 wks gestation (regression coefficient -0.36, SE 0.2, OR 0.70, 95% CI 0.49 to 0.99, P < 0.01).

**Figure 3:**
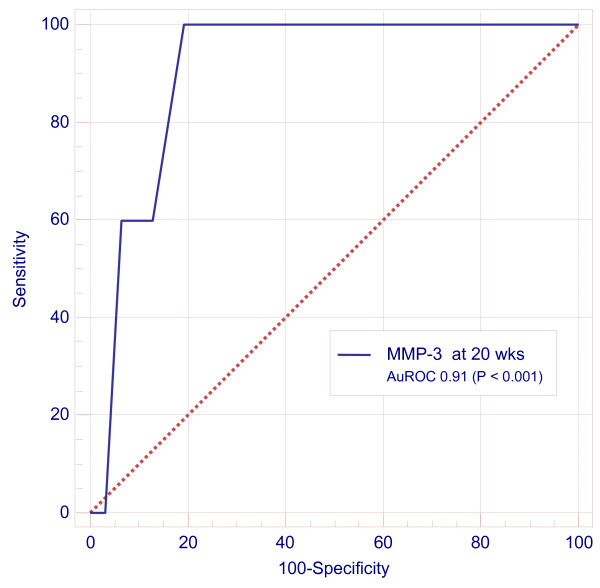
**Predictive association of MMP-3 with delivery before 37 weeks. ROC curve depicting predictive association between MMP-3 levels at 20 weeks gestation with delivery before 37 completed weeks**.

MMP-1, -3, -9, and TIMP-1 and -2 levels did not correlate with the average UA RI at any gestation. At all study gestations serum levels of MMPs and TIMPs were not significantly different in women who had prelabour rupture of membranes < 1 hour compared to women who went into labour with intact membranes. Furthermore, levels did not correlate with fetal birth weight and preterm delivery with one exception: the mean (SE) concentrations of MMP-3 at 20 weeks gestation were higher in women who delivered before 37 weeks gestation (8.8 ± 0.7 ng/ml) than those who delivered at term (4.7 ± 0.4 ng/ml, P < 0.01). Without regard to history of previous pregnancy loss, serum MMP-3 at 20 weeks gestation appeared predictive of delivery before 37 completed weeks (n = 5) with an AuROC of 0.91 (95% CI 0.76, 0.98, P < 0.001): serum MMP-3 > 6 ng/ml appeared to predict delivery before 37 weeks with a sensitivity of 100%, a specificity of 81%, and a +ve likelihood ratio of 5.2 (95% CI 4.3, 6.1), Figure 3.

Levels of studied proteins in cord blood did not differ between RPL and NRPL, and did not correlate with their antenatal levels, Doppler indices, fetal birth weight, gestational age at delivery or prelabour amniorrhexis.

## Discussion

We report, for the first time, that during a viable pregnancy, women with a history of RPL have raised serum levels of TIMP-2 whilst MMP-1, -3, and -9 are not significantly altered. We have also previously reported reduced serum RLX levels in these women, and our current observations suggest that both serum RLX and TIMP-2 concentrations discriminate between pregnant women with a history of RPL and those without. Expressed as a ratio, RLX-2:TIMP-2 < 0.1 at 10-12 weeks gestation was strongly associated with a history of RPL, achieving a sensitivity of nearly 100%, a specificity of 75% and a positive likelihood ratio of 4. Our observations suggest functional roles for these two peptides during early pregnancy development. However, our limited sample sizes precluded determination of any predictive relationship between these peptides and pregnancy loss, and adverse outcome in the index pregnancy, highlighting the need for larger scale prospective studies.

We have compared two groups of pregnant women that were matched for age and differed only with respect to their previous history of recurrent pregnancy loss. Consequently, differences in the serum concentrations of the study peptides between the two groups are likely to be attributable to their status with respect to a history of RPL. A raised TIMP-2 level in pregnant women with a history of RPL suggests that TIMP-2 plays a role in early pregnancy homeostasis, and that RPL may be associated with some dysregulation of TIMP-2. It is plausible that a critical level of increased TIMP-2 activity is required before a pregnancy fails, and that such critical level was not attained in the women with a history of RPL who achieved favourable outcomes during the index pregnancies. On the other hand, the observed rise in TIMP-2 levels in the RPL group may serve to sustain the at-risk pregnancy. Further studies are required to elucidate the physiological roles of TIMP-2 and relaxin during early pregnancy, and to determine whether the altered expression levels of TIMP-2 and relaxin-1 during pregnancy to women with a history of RPL contribute to the cause or are a consequence of recurrent pregnancy loss.

The levels of MMP-1 -3, and -9 are unaltered in RPL in comparison to NRPL. Serum TIMP-1 concentrations also did not differ with history of RPL, consistent with the thesis that TIMP-1 and TIMP-2 have distinct individual, perhaps tissue-specific, as yet ill-understood, biological functions.

Although originally characterized as inhibitors of MMP activity, TIMPs are known to have distinct MMP-independent functions in biological tissues, being involved in cell growth, migration and apoptosis [21,22]. TIMP-1 has adipokinetic effects [19] while TIMP-2 is a potent inhibitor of angiogenesis [23]. Recent studies have identified specific signaling pathways and cell surface binding partners for members of the TIMP family. TIMP-2 binding to the integrin α3β1 is the first description of a cell surface receptor for a TIMP family member [24,25]. TIMP-2 has also been shown to induce gene expression, promote G1 cell cycle arrest, and inhibit cell migration whilst TIMP-1 binding to CD63 inhibits cell growth and apoptosis [21]. These findings suggest that TIMPs play complex roles in controlling cell fate, acting directly through cell surface receptors and indirectly through modulation of protease activity [21].

TIMP-2 levels are elevated in the third trimester in women with impaired glucose tolerance and gestational diabetes [26]. Although reports are conflicting, some women with RPL (particularly those with PCOS) demonstrate insulin resistance [3,27-29]. Insulin resistance may therefore partly explain our observation of raised TIMP-2 levels in those women with a history of RPL. Stojanovic et al [23] demonstrated that TIMP-2, expressed as a ratio of MMP-2, was strongly correlated with impaired glucose tolerance. Furthermore, a murine model of hyperinsulinaemia and insulin resistance is associated with increased TIMP-1 and -2 levels, suggesting that insulin homeostasis may affect extracellular matrix metabolism via TIMP-mediated processes [30].

Our observation of elevated TIMP-2 levels in women with a history of RPL may suggest that functional dysregulation of this protein may contribute to early pregnancy failure by altering matrix remodeling at the implantation site (through regulation of proteinases) and inhibiting angiogenesis (through a direct effect at the choriodecidual interface). TIMP-2 inhibits fibroblast growth factor-stimulated proliferation of human endothelial cells via a MMP-independent signaling mechanism [31], consistent with this thesis. Control of cellular growth and differentiation by TIMPs via MMP-dependent regulation of insulin growth-factor binding protein availability has also been described [32].

To our knowledge, this is the first report of serum levels of representative MMPs and TIMPs during human pregnancy. MMP-2 and -9 serum levels are reported to be increased in women with pre-eclampsia [33-35]. Pro-MMP-9 levels increase whilst TIMP-1 levels decrease prior to labour [36], and following preterm amniorrhexis [37]. We could not confirm the latter observation in our cohort of women who had prelabour membrane rupture > 1 hour as our data sets were small and our last maternal serum samples were taken remote from term (at 34 weeks gestation).

An important caveat to our observations is that we have studied systemic rather than gravid tissue MMP and TIMP levels. We may therefore have missed more profound local changes associated with early pregnancy development or prelabour fetal membrane rupture in late pregnancy. Nonetheless, we still demonstrated dramatic differences in serum relaxin and TIMP-2 concentrations, suggesting that further studies may provide additional mechanistic insight into the biology of these molecules in human reproduction. The limitations of extrapolating serum levels of these molecules to their function in local reproductive tissue notwithstanding, it is still plausible that they may prove of predictive clinical value for adverse pregnancy outcomes such as miscarriage. Measuring serum levels of these proteases and their inhibitors are currently being investigated as preclinical and clinical markers of disease progression in advanced cancers of various organ systems [38].

Despite our limited sample sizes, we observed, for the first time, a strong association between high MMP-3 levels and delivery before 37 weeks gestation. This observation is in keeping with reports of increased levels of MMP-3 in the amniotic fluid and maternal decidua of women with chorioamnionitis and inflammation-induced preterm delivery [39,40]. Larger studies are required to confirm whether MMP-3 levels are elevated by 20 weeks gestation in women who deliver before 37 weeks as this intriguing observation could prove a useful adjunct to current screening tools for preterm delivery.

Our experimental model has several draw-backs and limitations. Firstly, several different tissues contribute to systemic levels of serum markers and local expression levels are likely to vary by tissue. The contribution of reproductive tract tissue to serum levels of MMPs and TIMPs during pregnancy is unknown and inferences regarding activity of these serum markers in gravid reproductive tract tissues may be somewhat spurious. However, we have compared age-matched cohorts of pregnant women with and without a history of RPL and our observed differences are therefore highly likely to reflect the underlying differences in their history of recurrent pregnancy loss. Secondly, our sample sizes were small and our study may have been under-powered to detect weaker associations of serum marker levels and the clinical outcome measures investigated. Larger scale prospective studies of cohorts with sufficient subpopulations of women with adverse pregnancy outcomes will be required to confirm or refute any potential predictive clinical utility of assessing serum relaxin and TIMP-2 levels. The miscarriage rates in the index pregnancy in our series were so low that we could not determine the temporal relationship, if any, between serum RLX-2 and TIMP-2 levels and subsequent miscarriage. This determination is the subject of ongoing studies.

Low serum relaxin appears to enhance the strong association of high TIMP-2 levels with a history of RPL, suggesting that these two peptides may regulate endometrial or placental angiogenesis through synergistic mechanisms, either directly or via the inhibition of angiogenic growth factors such as vascular endothelial growth factor (VEGF) and placental growth factor [41,42].

## Conclusions

We have demonstrated that serum TIMP-2 levels are increased during pregnancy to women with a history of RPL in comparison to pregnant controls with no history of RPL. Furthermore, when expressed as a ratio of serum relaxin, there is an enhanced separation at 10-12 weeks gestation, of women with a history of RPL from those without. We have also noted, despite a limited sample size, that raised serum MMP-3 levels at 20 weeks gestation appears predictive of delivery before 37 weeks. Taken together, our data suggest that dysregulated RLX-2 and TIMP-2 may play a role in habitual early pregnancy failure. Further prospective studies of larger cohorts are required to confirm or refute these observations, and to investigate any predictive clinical utility.

## Competing interests

The authors declare that they have no competing interests.

## Authors' contributions

All authors have contributed to and approved the final manuscript. DA conceived the study, took part in the clinical studies and wrote the manuscript. SE took part in the clinical and laboratory studies and read the manuscript. SLE took part in the laboratory studies and read the manuscript. TCL contributed ideas to the project and to the manuscript. All authors read and approved the final manuscript.
